# Modeling the Initiation of Others Into Injection Drug Use, Using Data From 2,500 Injectors Surveyed in Scotland During 2008–2009

**DOI:** 10.1093/aje/kwu345

**Published:** 2015-03-18

**Authors:** Simon R. White, Sharon J. Hutchinson, Avril Taylor, Sheila M. Bird

**Keywords:** incarceration rate, initiator characteristics, injection drug users, injector incidence, replacement rate

## Abstract

The prevalence of injection drug use has been of especial interest for assessment of the impact of blood-borne viruses. However, the incidence of injection drug use has been underresearched. Our 2-fold aim in this study was to estimate 1) how many other persons, per annum, an injection drug user (IDU) has the equivalent of full responsibility (EFR) for initiating into injection drug use and 2) the consequences for IDUs' replacement rate. EFR initiation rates are strongly associated with incarceration history, so that our analysis of IDUs' replacement rate must incorporate when, in their injecting career, IDUs were first incarcerated. To do so, we have first to estimate piecewise constant incarceration rates in conjunction with EFR initiation rates, which are then combined with rates of cessation from injecting to model IDUs' replacement rate over their injecting career, analogous to the reproduction number of an epidemic model. We apply our approach to Scotland's IDUs, using over 2,500 anonymous injector participants who were interviewed in Scotland's Needle Exchange Surveillance Initiative during 2008–2009. Our approach was made possible by the inclusion of key questions about initiations. Finally, we extend our model to include an immediate quit rate, as a reasoned compensation for higher-than-expected replacement rates, and we estimate how high initiates' quit rate should be for IDUs' replacement rate to be 1.

Understanding the health risks of injection drug users (IDUs), including injection-related transmission of blood-borne viruses, is of international importance. The prevalence of current IDUs has been of especial interest for assessment of the impact of blood-borne viruses on the injector population (1, 2). However, there has been less focus on studying IDU incidence per se, yet the IDU population is primarily sustained by the initiation of others. Large-scale IDU incidence studies to quantify the rate of initiations to injecting have been few, as these are hard to design well.

“[The] number of new initiates to injecting, in your presence, in the past year” was identified as an essential new question in *21st Century Drugs and Statistical Science in UK* (3, p. 19). Contemporaneously, a national cross-sectional community-based survey of over 2,500 IDUs in Scotland, which was conducted during 2008–2009, included just such a question.

The Scottish data allow us to analyze who initiates, shared responsibility for initiations, and the initiation rate in the past year for which an IDU has the equivalent of full responsibility (EFR).

Next, we consider the implied IDU population dynamics by, as have others (4–6) before us, treating injecting as a behaviorally transmitted epidemic into which individuals are inducted. Of interest is the so-called replacement-rate, *R*. Combining the EFR initiation rate and the injecting career length with different assumptions for the annual rate of cessation from active injecting of established IDUs, we define IDUs' replacement rate as the expected number of EFR initiations during a typical injecting career. If the replacement rate is equal to 1, then the IDU population will be stable, whereas a rate greater than 1 implies a growing IDU population, and less than 1 indicates that the IDU population shrinks over time.

Finally, to resolve data conflicts, we posit that there is an immediate “quit rate” for initiations that did not result in the novice becoming an active injector. We adapt our model and investigate how high such an immediate quit rate by novices would have to be for the IDU replacement rate to be equal to 1. Because *R* = 1 implies that each IDU is expected to replace him/herself by just 1 other, the number of current IDUs is not expected to increase, which is in line with the minimal growth in Scotland's IDU prevalence over the last decade.

## BACKGROUND OF SCOTLAND'S IDU EPIDEMIC

In the early 1980s, Scotland experienced an epidemic of injection drug use (7) and of injection-related blood-borne viruses, notably human immunodeficiency virus (8), hepatitis B virus (9), and hepatitis C virus (10) infections. Scotland was quick to invest in blood-borne virus prevalence studies (11–13) and in capture-recapture estimation of its current IDUs (14, 15), but incidence studies—whether of blood-borne viruses (16) or initiations to injecting (3)—were initially lacking.

## REVIEW OF APPROACHES TO ESTIMATING IDU INCIDENCE

Estimates of historical IDU incidence have been derived by reweighting samples of current IDUs with known duration of injecting (17 and, alternatively, by adjusting treatment data for the delay between onset of injecting and first referral to a treatment agency (18). Back-calculation applied to opiate overdose deaths to estimate trends in IDU incidence has been tried, on the basis that most opiate overdose deaths occur among IDUs (19). Expert epidemiologic opinion has also been called upon, as in Scotland (10), to give guesstimates for IDU incidence and cessation, as in Australia too (20). Transition studies into and out of injecting have also been reported (21–23).

## REVIEW OF PAST IDU INCIDENCE STUDIES

Hunt et al. (24, 25) considered interventions to dissuade IDUs in southeast England from initiating; 34 of 86 (40%) original participants (mean age = 30 years) had initiated 72 people throughout their injecting careers, but past-year initiations were not elicited.

Day et al. (26) surveyed 399 heroin users in Sydney, Australia (mean age at interview = 31 (standard deviation, 8.2) years; median injecting-career length, 9.5 years), of whom almost all had injected heroin. Self-initiation to injecting was reported by 10% (refer also to Doherty et al. (27)); 149 (37%) reported having ever taught someone to inject and had initiated a median of 3 (range, 1–200). One in 6 (17%) reported having taught someone to inject in the past year, the median being 2 novices (range, 1–50), which implied an initiation rate of 0.34 per IDU per annum.

Kermode et al. (28) recruited 200 early career IDUs (age at interview: mean = 24.5 (standard deviation, 2.2) years) in northeast India, 138 of whom (69%) had initiated 690 others over injecting careers of a mean length = 3.4 years, which implied an initiation rate of 1.01 per IDU per annum. However, in northeast India, initiation was described as overwhelmingly a social event, as 98% were with others at the time of their own initiation. On average, there were between 3 and 4 people present (including the person being initiated), so the 690 initiations may have related to many fewer initiates because of multiple counting by those present at the same time.

## METHODS

### Equivalent of full responsibility initiation rate

Participants in Scotland's Needle Exchange Surveillance Initiative (NESI) survey were asked 2 questions. 1) In the past year, how many times have you been present when someone injected for the first time, namely, at the initiation of novice injectors? Those who had been present for at least 1 initiation then were asked the second question. 2) How many other IDUs were present (respondent and novice excluded) at the most recent initiation in the past year? Important for what follows was the explicit request for the number of other IDUs present.

Using these responses, we defined a measure of initiations per IDU, which takes account of equally shared responsibility among those present at initiation events. Thus, the annual rate of initiations for which an initiator has the equivalent of full responsibility (EFR), λ_EFR_, is the sum of the proportion of responsibility over all initiations the IDU is present at in the past year:

λEFR=∑i=1numberofinitiationspresentatinpastyear (share of responsibility for the ithinitiation).

Assuming that the number of other IDUs present at the most recent initiation was either representative for the past year or constant over all initiations that the respondent was present at during the year, we divided the EFR rate (that is, the number of initiations) by the number of other IDUs present plus 1 (the added 1 is the respondent):

$$\lambda_{\mathrm{EFR}}=\frac{\text{number}\;\text{of}\;\text{initiations}\;\;\text{present}\;\text{at}\;\text{in}\;\;\text{past year}}{\begin{array}{c} \text{number}\;\text{of}\;\text{other}\;\text{IDUs}\;\;\text{present at most}\;\\ \text{recent}\;\text{initation}+1 \end{array}}.$$

Estimates of the overall EFR rate are computed by taking the mean over all individual EFR rates, including individuals with 0 initiations in the past year. Subgroup EFR rates were computed by restricting the sample to specified categories of individuals, so that the EFR rate can be thought of as a function of a limited set of observed covariates, which can be themselves time varying.

Bootstrap resampling was used to obtain 95% confidence intervals for the λ_EFR_ estimates. The pair, number of initiations present, and number of other IDUs also present were sampled for each observed individual, correctly accounting for the joint distribution of the component aspects.

### EFR rate regression analyses

We fit a weighted linear regression for λ_EFR_—or for a logarithmic transformation, ln(λ_EFR_), to reduce skewness and give a better fitting model—in R (R Foundation for Statistical Computing, Vienna, Austria) using the lm() function with the subgroups as covariates to investigate the effect of sex, injecting career, and ever incarcerated. We used the bootstrap precisions (reciprocal of the variances) as weights in the regression to account for the uncertainty in the subgroup estimates.

### Injectors' piecewise constant incarceration rates by sex

A priori, we expect incarceration to be associated with changes in initiation behavior, as it has be shown that initiations occur inside prison (29, 30) and that experience may alter an individual's initiation behavior. In the following, we show, using the EFR rate regression analysis, that NESI's EFR initiation-rates are strongly associated with incarceration history, so that our analysis of IDUs' replacement rate must incorporate when, in their injecting career, IDUs were first incarcerated.

We model an IDU's time at first incarceration because initiation into injecting, *X*, as a random variable using piecewise constant annual incarceration rates over 3 injecting-career-length intervals of 0–5, 6–10, and ≥11 years, denoted *p*_1_, *p*_2_, and *p*_3_, respectively.

The time of first incarceration, *X*, has the following (discrete) probability distribution:

$$P(X<\;x)=\left\{\begin{array}{cc} 1 & \text{if}\;x=0\\ {\left(1-\;p_{1}\right)}^{x} & \text{if}\;0<x\leq 5\\ {\left(1-\;p_{1}\right)}^{5}{\left(1-\;p_{2}\right)}^{x-5} & \text{if}\;5<x\leq 10\\ {\left(1-\;p_{1}\right)}^{5}{\left(1-\;p_{2}\right)}^{5}{\left(1-\;p_{3}\right)}^{x-10} & \text{otherwise}, \end{array}\right.$$

in which per annum incarceration rates feature as the stopping probability in a standard geometric distribution.

We can infer sex-specific incarceration rates using maximum likelihood techniques. For each NESI respondent, we know his/her injecting career length (in whole years) at survey and whether he/she has ever been incarcerated (self-reported). By sex, the likelihood of observing the NESI responses, assuming unbiased sampling, is

$$L(m_{1},\ldots ,m_{T},n_{1},\ldots ,n_{T})=\prod_{t=1}^{t=T}P{\left(X\leq t\right)}^{m_{t}}P{\left(X>t\right)}^{n_{t}},$$

where *m_t_* and *n_t_* are the counts of same-sex individuals with a career length of *t* years when surveyed who have ever (*m_t_*) versus never (*n_t_*) been in prison, respectively, and *X* is the time at first incarceration. The likelihood above is maximized in R by using the optimize() function, and confidence intervals were computed by using the profile likelihood method.

### Injectors' replacement rate

To compute the number of initiations during an injecting career, we must first consider the length of an injecting career, *IC*, itself a random variable. We assume a simple geometric distribution, with a per annum cessation rate, *c*, and a one-time immediate quit rate, *q*, such that *IC* has the probability distribution:

$$P(IC>t)=(1-q)(1-c{)}^{t}\;\;\text{for}\;t=0,1,2,\ldots \;.$$

With this injecting career distribution, the expected career length is (1 − *q*)/*c*.

“Cessation” is defined as the permanent stopping of injecting and no longer being responsible for any initiations. Thus, cessation includes individuals who truly cease injecting, as well as those individuals who die (drug-related deaths and other causes).

A new initiate quits if he/she ceases being an IDU immediately. Within our discrete time model, this implies 0 years as an IDU, and hence these individuals contribute no years as an injector and cannot themselves initiate anyone.

Our injector replacement rate, *R*, can be computed as follows:

(1) $$R=\sum_{w}\sum_{x}\sum_{t}\lambda_{\mathrm{EFR}}(t,x,w)P(IC>t)P(X=x),$$

where λ_EFR_(*t*, *x*, *w*) is the appropriate per annum EFR initiation rate for career length, *t*, given that the first incarceration event occurred at time *x* and with the covariate vector ***w*** (which is time invariant).

### Estimation of quit rates

To determine the quit rate, *q*′, which results in a replacement rate equal to 1, we observe that the quit rate parameter occurs only in 1 term in equation 1, and it can be taken outside the triple sum as a common factor. Hence, equation 1 can be rewritten as *R*(*p*, *c*, *q*) = (1 − *q*) *f* (*p*, *c*), where *f* corresponds to the triple summation over career length, covariates, and time of first incarceration with the (1 − *q*) factor removed. Our initial estimates of *R* assume that *q* = *c*, so we an calculate the value of *f* (*p*, *c*) and thus find the quit rate, *q*′, that makes *R* equal to 1 by rearranging as follows:

$$\begin{array}{rl} R(c,p,c) & =(1-c)\;f(\;p,c)\\ & \Rightarrow f(\;p,c)=\frac{R(c,p,c)}{1-c}\\ R(c,p,q^{\prime }) & =(1-q^{\prime })\;f(\;p,c)\\ 1 & =(1-q^{\prime })\;f(\;p,c)\\ 1 & =(1-q^{\prime })\frac{R(c,p,c)}{1-c}\\ q^{\prime } & =1-\frac{1-c}{R(c,p,c)}. \end{array}$$

All tabulations and analyses were performed in the GNU R statistical software (31).

## OVERVIEW OF NESI DATA

### Study population

A cross-sectional voluntary anonymous survey conducted from June 2008 to June 2009 recruited participants by using trained interviewers at 103 sites providing injecting equipment in mainland Scotland. To be eligible for interview, participants had to have injected at some time in their lives, but recruitment of individuals who had not injected in the past 6 months was limited to approximately one-fourth of the sample (refer to Web Appendix 1 available at http://aje.oxfordjournals.org/ for further details on the study population).

### Covariates

Participants were categorized by sex, region (Greater Glasgow and Clyde, elsewhere in Scotland), age group (<35, ≥35 years), self-reported length of injecting career (0–5, 6–10, 11–15, ≥16 years), and primary reason for attending the recruitment site (methadone, needle exchange, other-not otherwise specified). Scotland's needle exchanges provide equipment beyond clean needles/syringes.

Respondents were also asked whether they had ever been in prison or a young offenders' institution and, if yes, had they ever injected while incarcerated; latest hepatitis C virus test result; if ever prescribed methadone; if they had lived in a hostel in the last 6 months; and to recall their frequency of injecting in the months when they had injected in the last 6 months (Web Table 1).

### Exclusion of participants from analysis and minimally missing data

Of 2,563 participants, 27 (1%) did not respond to the primary classifiers of sex, region, age group, and reason for attendance and, as nonresponders, have been excluded from further consideration.

Of the 2,536 respondents, only 29 (1%) had missing data on at least 1 further key covariate used in regression analyses and so, without loss of generality, all regressions have been limited to the 2,507 respondents with complete covariate information. Of these, 450 (18%) were present at an initiation in the past year, only 7 of whom (2%) did not report how many other IDUs were present at their most recent initiation.

### Examination of the number of initiations and other injectors present

**Figure 1.**
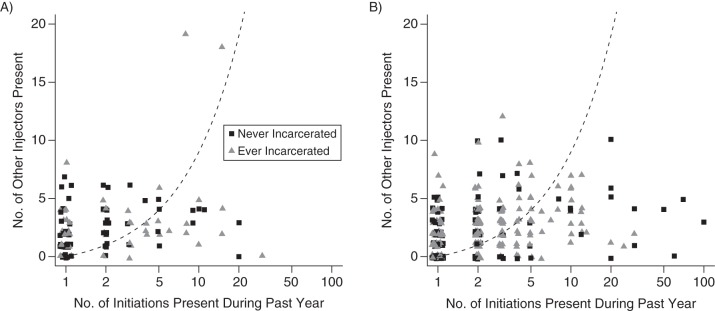
The number of initiations in the last year (on the log scale) by the number of other injectors present at the most recent initiation for the 112 female (A) and 331 male (B) respondents present for at least 1 initiation, NESI Study, Scotland, 2008–2009. The dotted line defines λ_EFR_ = 1, assuming equally shared responsibility. Explicitly, the line has the form, present = other IDUs concurrently present + 1, and is curved on the plot because of the log scale. The number of other injectors present excludes the initiator and initiate. IDU, injection drug user; NESI, Needle Exchange Surveillance Initiative.

Figure 1 plots the number of initiations against how many others were also present at the most recent event. In summary, 34% of 283 ever-incarcerated and 48% of 167 never-incarcerated past-year initiators reported having been present at 1 initiation only. Moreover, only 12% of 278 past-year ever-incarcerated initiators reported that no other IDU was present at the most recent initiation, whereas 24% so reported among 165 never-incarcerated past-year initiators.

Considering the social context of initiation, 179 of 299 (60%) past-year initiators who were present at 1 or 2 initiations reported 2 or more other IDUs present at the most recent initiation, as did 111 of 144 (77%) of those IDUs present at 3 or more past-year initiations. A fifth of all past-year initiators reported attendance at 4 or more initiations.

### Characteristics of initiators

In the past year, 18% of respondents (453 of 2,536) had been present at the initiation of a novice injector. Only 40 of 381 (10%) respondents who had not injected in the past year were present at an initiation, but 412 of 2,139 (19%) were present among past-year injectors. Being present at an initiation was more likely among those whose injecting career was short: 187 of 806 (23%) if career length was 0–5 years but 266 of 1,724 (15%) if longer than 5 years.

Logistic odds on being a past-year initiator were estimated (Web Table 2). Sex, age, and region were not ultimately significant for predicting whether a respondent was an initiator or not, whereas injecting career length, having injected in the past year, and hostel living in the past 6 months were, as was history of incarceration. Receipt of methadone in the past 6 months was not an extra deterrent to being an initiator nor was the initiator's self-reported hepatitis C virus infection.

## APPLICATION TO NESI DATA

### Estimated annual rate of initiations for which an initiator has the equivalent of full responsibility (λ_EFR_)

For each of 24 cross-classifications by sex, region, injecting career length (0–5, 6–10, ≥11 years), and whether ever incarcerated, 5,000 bootstrap resamples within each subgroup were used to derive the estimated annual EFR rate, λ_EFR_, and 95% confidence intervals as shown in Table 1. The overall annual EFR rate for the NESI respondents is 0.26 (95% confidence interval (CI): 0.20, 0.33), varying as low as 0.11 for never-incarcerated, older career injectors and as high as 0.46 for never-incarcerated, early career injectors.

**Table 1.** Expected Number of Initiations in the Last Year for Which Needle Exchange Surveillance Initiative Respondents Have the Equivalent of Full Responsibility With Bootstrap Confidence Intervals, NESI Study, Scotland, 2008–2009

Subgroup by Never/Ever in Prison/YOI	No. of Respondents	No. of Initiators	No. of Initiations	Expected Initiations per Annum	95% CI	Mean No. of Other Injectors Present	95% CI	Expected EFR Initiations per Annum	95% CI
All	2,500	443	1,694	0.68	0.55, 0.83	2.53	2.32, 2.74	0.26	0.20, 0.33
All									
Never	1,027	165	789	0.77	0.51, 1.10	2.36	2.04, 2.70	0.30	0.18, 0.47
Ever	1,473	278	905	0.61	0.51, 0.72	2.63	2.37, 2.92	0.22	0.17, 0.28
Male									
Never	596	103	613	1.03	0.58, 1.56	2.31	1.87, 2.79	0.41	0.21, 0.66
Ever	1,202	228	700	0.58	0.48, 0.70	2.54	2.29, 2.79	0.20	0.16, 0.25
Female									
Never	431	62	176	0.41	0.26, 0.60	2.44	1.98, 2.91	0.16	0.09, 0.28
Ever	271	50	205	0.76	0.45, 1.10	3.04	2.17, 4.15	0.30	0.13, 0.55
GGC									
Never	318	50	284	0.89	0.45, 1.53	2.32	1.67, 3.05	0.32	0.17, 0.50
Ever	604	103	325	0.54	0.40, 0.71	2.29	1.92, 2.80	0.22	0.14, 0.35
Else									
Never	709	115	505	0.71	0.40, 1.11	2.37	2.03, 2.76	0.30	0.14, 0.52
Ever	869	175	580	0.67	0.53, 0.83	2.83	2.51, 3.19	0.22	0.17, 0.28
Career length, years									
0–5									
Never	482	102	531	1.10	0.62, 1.77	2.23	1.85, 2.63	0.46	0.23, 0.81
Ever	318	82	233	0.73	0.53, 0.98	2.49	2.11, 2.91	0.26	0.18, 0.36
6–10									
Never	286	40	180	0.63	0.24, 1.29	2.90	2.16, 3.74	0.22	0.08, 0.42
Ever	440	69	219	0.50	0.33, 0.71	2.42	1.97, 2.90	0.22	0.11, 0.38
≥11									
Never	259	23	78	0.30	0.41, 0.51	2.00	1.19, 2.91	0.11	0.06, 0.17
Ever	715	127	127	0.63	0.49, 0.81	2.83	2.37, 3.35	0.21	0.16, 0.26

Abbreviations: CI, confidence interval; EFR, equivalent of full responsibility; Else, elsewhere in Scotland; GCC, Greater Glasgow and Clyde; NESI, Needle Exchange Surveillance Initiative; YOI, Young Offenders Institute.

### EFR rate regression analyses

We find that regional differences are negligible, so in Table 2 only the 12 nonregional subgroups of interest were used in the weighted linear regression to determine if and how sex, career length, and incarceration history influence the logarithm of the EFR initiation rate. We find that sex is not influential but that the EFR initiation rate is significantly lower for those whose injecting career is ≥11 years and increased if ever incarcerated.

**Table 2.** Unadjusted and Weighted Regression for (Log) λ_EFR_ Including All Respondents, NESI Study, Scotland, 2008–2009

Covariate	λ_EFR_	95% CI	Ln λ_EFR_	95% CI	Weighted Regression for Ln λ_EFR_ Coefficient (SE)	95% CI	*P* Value
Intercept					−1.96 (0.10)		
Sex							
Female (baseline)	0.22	0.12, 0.33	−1.51	−2.12, −1.11			
Male	0.27	0.20, 0.36	−1.31	−1.61, −1.02	0.29 (0.18)	−0.13, 0.71	0.150
Career length, years							
0–5 (baseline)	0.38	0.23, 0.57	−0.97	−1.47, −0.56			
6–10	0.22	0.13, 0.34	−1.51	−2.04, −1.08	−0.64 (0.31)	−1.37, 0.10	0.081
≥11	0.18	0.14, 0.22	−1.71	−1.97, −1.51	−0.55 (0.15)	−0.90, −0.20	0.007^a^
Prison							
Never (baseline)	0.30	0.18, 0.47	−1.20	−1.71, −0.76			
Ever	0.22	0.17, 0.28	−1.51	−1.77, −1.27	0.70 (0.13)	0.40, 1.00	<0.001^a^

Abbreviations: CI, confidence interval; Ln, logarithm; NESI, Needle Exchange Surveillance Initiative; SE, standard error.

^a^ Indicates a significant *P* value.

### Injectors' piecewise constant incarceration rates by sex

**Figure 2.**
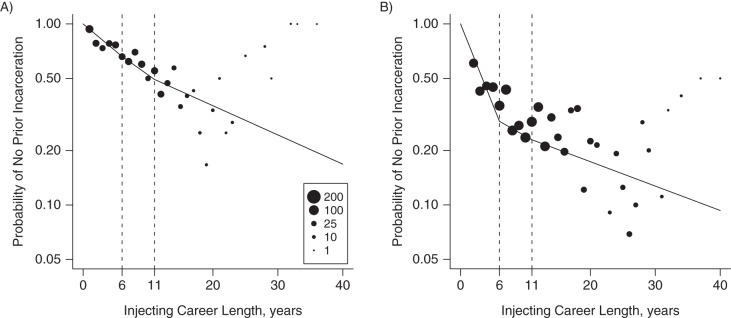
For females (A) and males (B), the logarithm of the proportion of respondents who have never been incarcerated during their injecting career versus injecting career length (in whole years), NESI Study, Scotland, 2008–2009. The size of each point denotes the number of respondents. On the logarithmic scale, the plot should be monotonically decreasing; the errant points on the right side are due to the small number of respondents. We modelled these data as piecewise constant per annum incarceration rates over 3 periods. The fitted lines are obtained from the maximum likelihood estimates in Table 3; the 3 line segments correspond to the piecewise constant periods for 0–5, 6–10, and ≥11 years, respectively. NESI, Needle Exchange Surveillance Initiative.

Table 3 gives the maximum likelihood estimates and 95% confidence intervals for the per annum incarceration rates and plotted as cumulative probability of incarceration in Figure 2. For female IDUs, we see a fairly constant incarceration rate per annum over their injecting career, namely: 7% (95% CI: 6, 8) per annum for the first 5 years, 6% for the second, and 4% thereafter. For males, by contrast, we derive a very high incarceration rate of 19% (95% CI: 17, 20) per annum for the first 5 years. However, thereafter, the incarceration rate for males reduces to 5% per annum for the second quinquennium and 3% thereafter. The incarceration rates in Table 3 confirm a significant difference between females and males in the first 5 years, but not beyond.

**Table 3.** Maximum Likelihood Estimates for the Piecewise Per Annum Constant Incarceration Rates, NESI Study, Scotland, 2008–2009

Sex	Length of Injecting Career, years					
	0–5		6–10		≥11	
	Probability	95% CI	Probability	95% CI	Probability	95% CI
Female	0.07	0.06, 0.08	0.06	0.04, 0.08	0.04	0.01, 0.06
Male	0.19	0.17, 0.20	0.05	0.02, 0.06	0.03	0.02, 0.05
Overall	0.14	0.13, 0.15	0.05	0.04, 0.07	0.04	0.03, 0.05

Abbreviations: CI; confidence interval; NESI, Needle Exchange Surveillance Initiative.

### Injectors' injecting-career length

The distribution of injecting-career lengths for Scotland cannot be reliably inferred from NESI respondents because of sampling biases. Specifically, although current IDUs were proportionally sampled, the maximum ratio was predetermined of current IDUs to those who had not injected in the past 6 months (but were sampled because of their attendance for needle exchange or methadone).

Instead, we have assumed a common cessation rate of *c* per annum (implying a geometric distribution) and an initial quit rate of *q* for initiations that did not result in active injecting. We consider 3 plausible scenarios defined by commonly assumed cessation rates of 5%, 10%, or 15% per annum (10, 32, 33), with the quit rate initially set equal to the annual cessation rate.

### Injectors' replacement rate: quit rate set equal to annual cessation rate

Table 4 shows the replacement rates, *R*(*c*), computed by using equation 1 with the estimated piecewise constant per annum incarceration rates *p*_1_, *p*_2_, and *p*_3_, cessation scenarios in which the quit rate is initially set equal to the cessation rate, and the 12 cross-classified EFR initiation rates. Replacement rates were derived separately for female and male IDUs as well as overall, because incarceration rates were significantly different by sex.

**Table 4.** Expected Number of Career Total Initiations (the Replacement Rate), NESI Study, Scotland, 2008–2009

Sex	Cessation Rate					
	0.05		0.10		0.15	
	Replacement Rate	95% CI	Replacement Rate	95% CI	Replacement Rate	95% CI
Female	3.7	2.4, 5.2	1.8	1.2, 2.6	1.1	0.8, 1.6
Male	5.5	3.9, 7.4	3.3	2.1, 4.8	2.4	1.4, 3.8
Overall	4.8	3.7, 6.1	2.7	1.9, 3.8	1.9	1.3, 2.7

Abbreviations: CI; confidence interval; NESI, Needle Exchange Surveillance Initiative.

With the quit rate set equal to the cessation rate, our estimates of *R*(*c*) are high even for a substantial cessation rate of 15% per annum. By sex, central estimates of *R*(*c*) are greater than 1 and significantly so for males at 2.4 (95% CI: 1.4, 3.8) versus 1.1 (95% CI: 0.8, 1.6) for females.

### Estimation of quit rates

Our EFR initiation rate of 0.26 (95% CI: 0.20, 0.33) would suggest that, if injecting careers averaged 5–10 years or more and all initiated novices embarked on an injecting career, then IDUs' replacement rate would greatly exceed 1.

Thus, we estimated the quit rates under each cessation scenario, such that *R* = 1. For male IDUs, the quit rates had to be very high, with central estimates greater than 50% for all cases in Table 5. For females, given the lower number of initiator respondents in NESI, there is more uncertainty but, as for the males, the estimated quit rate is higher than the associated cessation rate.

**Table 5.** Required Quit Rate to Maintain a Stable Injection Drug User Population, NESI Study, Scotland, 2008–2009

Sex	Cessation Rate					
	0.05		0.10		0.15	
	Quit Rate	95% CI	Quit Rate	95% CI	Quit Rate	95% CI
Female	0.74	0.60, 0.82	0.51	0.25, 0.65	0.25	0.00, 0.47
Male	0.83	0.76, 0.87	0.73	0.56, 0.81	0.65	0.39, 0.78
Overall	0.80	0.75, 0.85	0.67	0.53, 0.76	0.55	0.33, 0.70

Abbreviations: CI; confidence interval; NESI, Needle Exchange Surveillance Initiative.

If the annual cessation rate for Scotland's established IDUs were 10% or lower, then even the lower 95% confidence limit for novices' quit rate had to be greater than 50% for there to be no more than 1-for-1 replacement of IDUs. Central estimates (to the nearest 5%) paired the 80% immediate quit rate with the 5% annual cessation rate by established injectors, the 70% immediate quit rate with the 10% cessation rate, and the 55% immediate quit rate with the 15% cessation rate.

## DISCUSSION

We have developed a model for IDU initiation and IDUs' replacement rate that accounts for shared responsibility and have demonstrated the approach using survey data on Scotland's population of IDUs. This was possible because of the inclusion of basic questions in NESI asking how many other IDUs were also present at the most recent initiation event each respondent attended.

### Sampling bias in the NESI data

We compared the demography of the participants in NESI, 2008–2009, with the Bayesian estimates from Scotland's third capture-recapture study for current IDUs in 2009 (Web Table 3) and found strong agreement not only by region, which was by design, but also by sex and age.

The NESI sample is not necessarily representative of current and former injectors, however, as the proportion of former IDUs (defined as not having injected in the past 6 months) was predetermined. When we performed a sensitivity analysis reweighting the sample to be closer to Scotland's overall ratio of current to former IDUs (1:3 or 1:4), there was no important impact on the central estimates, which have broad confidence intervals.

The NESI sample was structured such that one-fourth had not injected in the past 6 months. It is difficult, of course, accurately to define when the transition from current IDU to former IDU occurs. We note that some of those who reported not having injected in the past year were involved in initiating, which is why our main analysis of ln λ_EFR_ retains these respondents (Web Appendix 2; Web Table 4). To assess the sensitivity of our results, Table 6 repeats the analysis after excluding 377 IDUs who had not injected in the past year (effectively setting their responsibility to 0); the covariate coefficients are slightly dampened but remain.

**Table 6.** Unadjusted and Weighted Regression for (Log) λ_EFR_ Excluding Respondents Who Had Not Injected in the Past Year, NESI Study, Scotland, 2008–2009

Covariate	λ_EFR_	95% CI	Ln λ_EFR_	95% CI	Weighted Regression for Ln λ_EFR_ Coefficient (SE)	95% CI	*P* Value
Intercept					−1.92 (0.14)		
Sex							
Female (baseline)	0.25	0.15, 0.41	−1.39	−1.90, −0.89			
Male	0.30	0.22, 0.41	−1.20	−1.51, −0.89	−0.03 (0.16)	−0.37, 0.30	0.837
Career length, years							
0–5 (baseline)	0.41	0.25, 0.63	−0.89	−1.39, −0.46			
6–10	0.25	0.14, 0.41	−1.39	−1.97, −0.89	−0.40 (0.20)	−0.82, 0.03	0.057
≥11	0.21	0.16, 0.27	−1.56	−1.83, −1.31	−0.41 (0.16)	−0.75, −0.07	0.028
Prison							
Never (baseline)	0.35	0.21, 0.53	−1.05	−1.56, −0.64			
Ever	0.24	0.19, 0.32	−1.43	−1.66, −1.14	0.56 (0.14)	0.26, 0.86	0.001^a^

Abbreviations: CI, confidence interval; Ln, logarithm; NESI, Needle Exchange Surveillance Initiative; SE, standard error.

^a^ Indicates a significant *P* value.

Our finding that 18% of over 2,500 NESI injectors in Scotland in 2008–2009 were present at an initiation in the past year agrees remarkably well with the 17% rate reported by Day et al. (26) for 399 heroin users (mostly injectors) in Sydney, Australia.

### Model assumptions

We use a discrete time model for cessation and incarceration, partly because the data are reported in whole years, but also so that parameters are interpretable as rates per annum.

In Web Appendix 3, we consider unequally shared responsibility, showing that, when averaged over all the IDUs present, preferential responsibility by an unknown member of those IDUs present reduces to our original assumption of equally shared responsibility. Thus, lacking any information on concurrently present IDUs, our assumption of equally shared responsibility is not only a reasonable one but also the statistically coherent approach.

For parsimony in the original 2008–2009 NESI surveillance, supplementary questions were not posed about self-initiation, sex of initiated, or persistence of the initiate's injecting career. The social, rather than numerical, context of initiations was also not explored. For example, we do not know the sex, prison history, and injecting career length of IDUs present at the same time.

Given the very different EFR rates for females and males, the IDU population dynamics will strongly depend upon the distribution of novices' sex, which most likely depends upon the sex of the initiator. However, there are no data within NESI, and there is a lack of other evidence to address this issue properly.

### Quit rate as an explanation for high overall EFR rate

Our initial cessation model was a discrete-time geometric model with a single per annum cessation rate parameter. However, our surprising discovery was that the estimated overall rate of EFR initiations is too high, given that Scotland's number of current IDUs has not been increasing exponentially in the 21st century. Bayesian capture-recapture estimates by Overstall et al. (34) of Scotland's IDU prevalence in 2000, 2003, and 2006 were 16,400 (95% CI: 14,200, 20,600), 22,900 (95% CI: 19,000, 27,800), and 15,700 (95% CI: 11,800, 18,700), respectively, showing no significant increase in the IDU population.

Explanations of our high EFR rate include the following: 1) IDU instructor thinks he/she is initiating but, in fact, the novice has had previous initiations; 2) systematic bias (the number of other IDUs present) is underestimated; 3) recall bias (the number of initiations attended in the past year) is exaggerated; 4) mismatch between NESI respondents and Scotland's current IDUs; 5) changes over time (initiation rates derived from IDUs' responses in 2008–2009 may differ from those that applied in the 1980s and 1990s), which gave rise to Scotland's number of current IDUs; and 6) many 21st century novices do not persist with injecting beyond the initiation event.

Explanations 1–3, around recall biases or deliberate misleading of IDU initiator by novices, are insufficiently plausible. We have already assessed the validity of generalizing from NESI respondents to Scotland's IDUs, and thus explanation 4 is also unlikely. Explanation 5 does raise the question of changing initiation and quit rates, and although the latter may have changed, it seems unlikely that the initiation rate would have changed so dramatically.

We therefore follow explanation 6 and have posited that many initiations fizzle out (that is, there is rapid desistence from injecting). Rapid desistence is not, of course, a theme that the surveying of established IDUs about their own initiation can elucidate and so needed to be estimated indirectly, as here. Sweeting et al. (33) proposed a method for estimating the prevalence of former and current IDUs and reported a substantial 34% (95% CI: 20, 50) proportion of former injectors, who had injected for only a short period, which they defined as less than 1 year. Our estimate, which has initiates as the denominator, is at least consistent with this alternative analysis given that *q* is more than 3 times *c*.

If our posited immediate quit rate is true, then there may be many individuals who had injected, in effect, once only and yet are typically not counted as former IDUs. This has implications for estimates of the number of ever-IDUs who may have been exposed, albeit by infrequent injection, to blood-borne viruses such as hepatitis C virus.

### Conclusions

We have presented a novel approach for estimating IDU incidence in terms of EFR initiation rates. The methodology we have presented can be applied to any representative survey of IDUs wherein respondents are asked about sex, incarceration history, injecting-career length, past-year initiations, and number of other IDUs simultaneously present. New NESI questions have been inspired by the current analysis, not only on novice cessations and persistence but also on prison initiation of respondents and the role that alcohol may play in encouraging initiations or in biased recall of the circumstances that prevailed, including the number of simultaneously present IDUs.

A by-product of our methodology has been the piecewise constant estimation of annual incarceration rates by duration of injecting career, separately for female and male IDUs. This has not been previously available for Scotland, and other jurisdictions may find it useful to compute their own national estimates.

The EFR incidence rates estimated via NESI constitute one of the largest IDU incidence studies to date, against which comparison can be made by future Scottish and international studies.

In summary, of 2,536 recruited IDUs in Scotland (mean age = 33.4 (standard deviation, 7.0) years), 453 respondents reported 1,721 initiation events in the past year (median, 2; range, 1–100) that, accounting for shared responsibility, implied an overall EFR initiation rate of 0.26 per IDU per annum.

## Supplementary Material

Web Material
