# Supplementary material for: Modeling the Initiation of Others Into Injection Drug Use, Using Data From 2,500 Injectors Surveyed in Scotland During 2008–2009
Source: Am J Epidemiol. 2015 Mar 18;181(10):771–80. doi: 10.1093/aje/kwu345 (PMC4423524; doi:10.1093/aje/kwu345)
Supplement: Web Material [file supp_181_10_771__index.html]

Modeling the Initiation of Others Into Injection Drug Use, Using Data From 2,500 Injectors Surveyed in Scotland During 2008–2009 — Web Material 

# Modeling the Initiation of Others Into Injection Drug Use, Using Data From 2,500 Injectors Surveyed in Scotland During 2008–2009

## Web Material

Web Material

**Files in this Data Supplement:**

- Web Material - Pdf file
