# Supplementary material for: Modeling the Initiation of Others Into Injection Drug Use, Using Data From 2,500 Injectors Surveyed in Scotland During 2008–2009
Source: Am J Epidemiol. 2015 Mar 18;181(10):771–80. doi: 10.1093/aje/kwu345 (PMC4423524; doi:10.1093/aje/kwu345)
Supplement: Web Material [file supp_kwu345_kwu345supp.pdf]

Web Appendix 1

Characterization of the Study Population

Sample characteristics

In a cross-sectional, voluntary anonymous survey, known as the Needle Exchange Surveillance Initiative (NESI), conducted during June 2008 to June 2009, participants were recruited by trained interviewers at 103 sites providing injecting equipment in mainland Scotland. Besides being willing to take part in NESI, chosen sites had to have a private room in which interviews could be conducted. To be eligible for interview, participants had to have injected at some time in their lives, but recruitment of individuals who had not injected in the past six months was limited to approximately one-quarter of the sample. This limitation was to maximise the sample of current injecting drug users (IDUs) for estimation of the incidence of HCV infection. By design, the number of IDUs to be recruited in each of the eleven NHS Board areas in mainland Scotland was proportional to Scotland's estimated numbers of current IDUs in 2003, the latest available counts when the survey was planned. Consented participants responded to 50 demographic/behavioural questions, and provided a blood spot sample for linked anonymous blood-borne virus (BBV) testing. Participants were not rewarded nor were they asked to recruit others, all eligible participants presenting at the sites were invited to take part in the survey. By request, data from a subset of questions were made available, as shown in **Web Table 1**.

Summary statistics

Web Table 1 characterises a) all 2536 respondents, who are then cross-tabulated by b) primary reason for attendance (881 for methadone, 1319 for needle exchange (NE)), and c) whether the respondent was present at an initiation in the past year (453).

Eighty-five percent of respondents had injected in the past-year and 80% in the last six months. In terms of primary characteristics, 28% were female, 37% resided in Greater Glasgow and Clyde (GGC), 38% were 35 years of age or older, 32% were in the first five years of their injecting career, and 59% had ever been in prison or YOI. Three-quarters of participants had been hepatitis C virus (HCV) tested, but 8% of testees (154/1890) did not know their HCV test-result. Last known status was HCV antibody positive for 38% of those who knew their test result (651/1736).

All but 14% of respondents had, at some time, been prescribed methadone, and 71% had been prescribed methadone in the past six months, including 56% (710/1319) of those attending primarily for NE. Even among those attending primarily for methadone, 59% (516/881) had injected in the past six months, of whom 48% had injected at least daily in their injector-months (248/516) compared to 69% at-least-daily injectors of those attending primarily for NE who had injected in the past six months (885/1287). Also, 27% of participants had lived in a hostel in the past six months.

Web Table 1 includes the subset of questions requested for analysis from among the original 50 questions on the NESI survey. Age, region and career length categories were defined prior to receiving the data based on previous work.

Web Table 1: (a) Characteristics of all NESI interviewees, and cross-tabulations of characteristics according to (b) primary reason for attending recruitment setting and (c) report of having been present at initiation of a novice injector in the last year. Percentages are given according to either the row '()' or column '[]' marginal totals, as relevant. NR=non-responses, GGC=Greater Glasgow and Clyde, YOI=Young Offenders Institute

**Selection:**	**(a)**		**(b)**				**(c)**	
**COVARIATES**	**All**		**Primary reason for attendance**				**Present at initiation in last year?**	
	**N= 2536**		**Methadone n = 881 (35%)**		**Needle Exchange n = 1319 (52%)**		**Yes n= 453 (18%)**	
**Gender:**								
Female	710	[28%]	302	(43%)	305	(43%)	113	(16%)
Male	1826	[72%]	579	(32%)	1014	(56%)	340	(19%)
**Region**								
Else	1603	[63%]	491	(31%)	960	(60%)	297	(19%)
GGC	933	[37%]	390	(42%)	359	(38%)	156	(17%)
**Age-group (years)**								
<35	1564	[62%]	515	(33%)	845	(54%)	302	(19%)
35+	972	[38%]	366	(38%)	474	(49%)	151	(16%)
**Injecting career (years)**								
00-05	809	[32%]	225	(28%)	481	(59%)	187	(23%)
06-10	741	[29%]	260	(35%)	370	(50%)	113	(15%)
11-15	522	[21%]	197	(38%)	255	(49%)	73	(14%)
16+	464	[18%]	199	(43%)	213	(46%)	80	(17%)
**Ever in prison/YOI**	NR = 3		NR = 2		NR = 0		NR = 0	
No	1040	[41%]	336	(32%)	584	(56%)	168	(16%)
Yes	1493	[59%]	543	(36%)	735	(49%)	285	(19%)
***IF YES*, ever injected in prison?**	NR = 7		NR = 3		NR = 3		NR = 1	
No	1322	[89%]	472	[88%]	654	[89%]	246	(19%)
Yes	164	[11%]	63	[12%]	77	[11%]	38	(23%)
**Latest Self Reported HCV status**	NR = 0							
Tested, but status not known	154	[6%]	55	[6%]	74	[6%]	34	[8%]
HCV –ve	1085	[43%]	330	[37%]	610	[46%]	189	[42%]
HCV +ve	651	[26%]	278	[32%]	285	[22%]	109	[24%]
Never tested	646	[25%]	218	[25%]	350	[27%]	121	[27%]
**Ever prescribed methadone**	NR = 0							
No	354	[14%]	1		310	[24%]	89	(25%)
Yes	2182	[86%]	880		1009	[76%]	364	(17%)
**Prescribed methadone in last 6 mths**	NR = 0							
No	725	[29%]	7		609	[46%]	150	(21%)
Yes	1811	[71%]	874		710	[54%]	303	(17%)
**Injection frequency in months injected during last 6 months**	NR = 0							
Less than once per day	760	[37%]	268	[52%]	402	[31%]	131	(17%)
At least daily	1277	[63%]	248	[48%]	885	[69%]	260	(20%)
No injecting in past 6 months	499		365		32		62	
**Lived in hostel in last 6 months**	NR = 2		NR = 1		NR = 0		NR = 1	
No	1846	[73%]	645	(35%)	980	(53%)	298	(16%)
Yes	688	[27%]	235	(34%)	339	(49%)	154	(22%)
**Injected in the last year**	NR =10		NR = 3		NR = 7		NR = 1	
No	382	[15%]	282	[32%]	24	[2%]	40	(10%)
Yes	2144	[85%]	596	[68%]	1288	[98%]	412	(19%)
**Present at how many initiations?**	NR = 6		NR = 4		NR = 1			
ZERO	2077	[82%]	743	[84%]	1049	[80%]	0	[0%]
1	176	[7%]	50	[6%]	111	[8%]	176	[39%]
2	129	[5%]	37	[4%]	81	[6%]	129	[28%]
3	51	[2%]	16	[2%]	27	[2%]	51	[11%]
4+	97	[4%]	31	[4%]	50	[4%]	97	[21%]
**At most recent initiation, how many other injectors were present (besides you & novice)**	NR = 7		NR = 2		NR = 5		NR = 7	
ZERO	73	[16%]	15	[11%]	51	[19%]	73	[16%]
1	80	[18%]	20	[15%]	56	[21%]	80	[18%]
2	102	[23%]	19	[14%]	66	[25%]	102	[23%]

3	71	[16%]	31	[23%]	33	[12%]	71	[16%]
4	63	[14%]	24	[18%]	32	[12%]	63	[14%]
5	22	[5%]	6	[5%]	13	[5%]	22	[5%]
6+	35	[8%]	17	[13%]	13	[5%]	35	[8%]

Web Table 2: Adjusted odds on being an initiator – that is: present at the initiation of at least one new injector within the last year. Model A includes only demographic covariates, model B adds injecting career-length, and model C includes all covariates. Likelihood ratio test for model B over model A, chi-squared on 3 degrees of freedom=20.9, p<0.001; for model C over model B, chi-squared on 6 degrees of freedom-25.6, p<0.001. CI = confidence interval.

**COVARIATES**	Single-factor regression (unadjusted odds)[b]		Multi-factorial regression (adjusted odds)[b]		
	Odds ratio	95% CI	Odds ratio	95% CI	p-value[a]
Basic logistic regression model: A					
Intercept			0.21	(0.17, 0.26)	
Male (baseline Female)	1.21	(0.96, 1.54)	1.26	(1.00, 1.60)	*0.05*
GGC (baseline Elsewhere)	0.88	(0.71, 1.09)	0.92	(0.74, 1.14)	0.42
Age 35+ (baseline <35)	0.77	(0.62, 0.95)	0.76	(0.60, 0.94)	*0.01*
Logistic regression model: B					
Intercept			0.27	(0.21, 0.34)	
Male	1.21	(0.96, 1.54)	1.29	(1.02, 1.64)	*0.04*
GGC	0.88	(0.71, 1.09)	0.93	(0.75, 1.16)	0.52
Age 35+	0.77	(0.62, 0.95)	0.77	(0.59, 0.99)	*0.04*
Career 06-10 (baseline 0-5)	0.60	(0.46, 0.78)	0.61	(0.47, 0.79)	*< 0.001*
Career 11-15	0.55	(0.40, 0.73)	0.57	(0.42, 0.76)	*< 0.001*
Career 16+	0.70	(0.52, 0.94)	0.82	(0.58, 1.16)	0.26
Final logistic regression model: C					
Intercept			0.14	(0.09, 0.22)	
Male	1.21	(0.96, 1.54)	1.15	(0.90, 1.49)	0.26
GGC	0.88	(0.71, 1.09)	0.94	(0.75, 1.17)	0.56
Age 35+	0.77	(0.62, 0.95)	0.81	(0.62, 1.05)	0.11
Career 06-10	0.60	(0.46, 0.78)	0.61	(0.46, 0.79)	*< 0.001*
Career 11-15	0.55	(0.40, 0.73)	0.56	(0.40, 0.76)	*< 0.001*
Career 16+	0.70	(0.52, 0.94)	0.80	(0.55, 1.15)	0.22
Ever in prison/YOI (baseline: never in prison/YOI)	1.22	(0.99, 1.51)	1.31	(1.04, 1.66)	*0.02*
Self reported HCV positive (baseline: HCV negative, status not known or not tested)	0.90	(0.70, 1.13)	1.03	(0.79, 1.33)	0.84
Injected in the past-year (baseline: no injecting in past year)	2.07	(1.48, 2.98)	1.79	(1.26, 2.60)	*0.002*
Lived in hostel in last 6 months (baseline: not lived in hostel in last 6 months)	1.50	(1.20, 1.86)	1.34	(1.06, 1.67)	*0.013*
Methadone in last 6 months (baseline: not prescribed)	0.78	(0.62, 0.97)	0.92	(0.73, 1.16)	0.46

[a] Italics indicates a significant p-value

[b] We fit logistic regression in R using the glm() function to investigate the log-odds on being an initiator.

Web Table 3: Comparing NESI population to IDU population estimates in 2009 from Bayesian capture-recapture analysis (1). Regional marginal totals are similar by design, but the matching of proportions by sex and age-group was not pre-determined in the NESI sampling protocol.

Demographic sub-group		Study	NESI's injectors: complete-case respondents		Scotland's injectors in 2009	
**Sex**	**Region**	**Age**	Number	%	Number	%
Female	Else	< 35	337	[13%]	2600	[17%]
		35+	110	[4%]	600	[4%]
	GGC	< 35	168	[7%]	800	[5%]
		35+	87	[3%]	300	[2%]
Male	Else	< 35	725	[29%]	5600	[36%]
		35+	411	[16%]	2700	[17%]
	GGC	< 35	315	[13%]	1700	[11%]
		35+	354	[14%]	1300	[8%]
**TOTALS**			2507		15700	

Web Appendix 1 References

1. Overstall A, King R, Bird SM, Hutchinson SJ, Hay G. Estimating the number of people who inject drugs in Scotland using multi-list data with left censoring. Under submission. 2012

Web Appendix 2

Characterization of Respondents Who Had Not Injected in the Past Year

Accurately defining a former-IDU is non-trivial. Injecting is a relapsing-remitting condition, so that injectors may go through several periods of off-injecting before truly ceasing. NESI respondents were asked whether they had injected in the past-year. The main analysis relates to all respondents, whether they had injected in the past-year or not. **Web Table 4** shows that about 1 in 11 of those who had not injected in the past-year were nonetheless present at initiations. Evidence from NESI respondents contradicts the belief that those who had not injected in the past-year were no longer involved in the initiation of novices.

Web Table 4: Cross-tabulation of those who had injected in the past-year against those who were present at an initiation in the past-year.

	Present at initiation in past-year		
**Injected in past-year**	**Yes**	**No**	**Total**
**Yes**	404 (19%)	1719 (81%)	2123
**No**	39 (10%)	338 (90%)	377
**Total**	443 (18%)	2057 (82%)	2500

Web Appendix 3

Shared Responsibility

Derivation of EFR rate

We define our EFR-initiation rate as the number of initiations an IDU has the equivalent of full responsibility for per annum. For each initiation which a respondent is present at, the respondent carries part of the responsibility.

The EFR-rate is defined as:

$$\lambda_{EFR} = \sum_{i=1}^{\substack{\text{number of initiations}\\ \text{present at in past-year}}} \text{(Share of responsibility for the } i^{\text{th}} \text{ initiation)}.$$

To be explicit, the total responsibility among all IDUs present at an initiation must sum to one.

Further, in full generality, the responsibility of any individual present may range from zero if they have no responsibility to one if they are wholly responsible.

Equally shared responsibility

In the main paper, we assume equally-shared responsibility among all IDUs present at an initiation.

NESI respondents were asked how many initiation-events were they present at, which does not convey any indication of the respondent's level of participation at each event. The reported number of other IDUs co-present at the most recent initiation is assumed constant/representative over all past-year initiations.

Our assumptions imply that an IDU has responsibility of $1/\text{Others}+1$ for each initiation at which they were present in the past-year. Specifically, responsibility is equally-shared among all IDUs present at each initiation. Thus we obtain

$$\begin{aligned}\lambda_{EFR} &= \text{(number of initiations present at in past-year)} \times \frac{1}{\text{(Others co-present)} + 1} \\ &= \frac{\text{Present}}{\text{Others}+1}.\end{aligned}$$

A reasonable question to consider is the effect of non-equally-shared responsibility, with greater responsibility assigned to a subset of those present, or to a single individual as the primary initiator. However, it is not possible to determine if the NESI respondent was the primary initiator for all or any of the initiations they were present at in the past-year, and so our assumption of equal-responsibility is the most neutral assumption given the data.

Example of non–equally shared responsibility

Consider the following adjustment to our EFR-rate. First, we assume that each NESI respondent is the primary initiator at each initiation, and that they will have a greater share of the responsibility.

For example, consider a NESI respondent who was present at 4 initiations in the past-year, and there were 2 other IDUs co-present on each occasion. Assuming equally-shared responsibility, the NESI respondent has an EFR-rate of:

$$\lambda_{EFR} = \frac{1}{3} + \frac{1}{3} + \frac{1}{3} + \frac{1}{3} = \frac{4}{3},$$

as there were three equally responsible IDUs at each of the four initiations.

If we now assume that the NESI initiator has 3/5th responsibility and the two other co-present IDUs for a 1/5th each per initiation, then the EFR-rate for the NESI initiator would be:

$$\lambda_{EFR} = \frac{3}{5} + \frac{3}{5} + \frac{3}{5} + \frac{3}{5} = \frac{12}{5}$$

Effectively, we are up-weighting the responsibility of the NESI respondent.

Randomly assigning primary initiator among those present

There is, however, no evidence to justify the assumption that the NESI respondent would be the primary initiator (that is: have a greater share of responsibility for the initiation) of each initiation at which they were present.

Assume there is a single primary initiator at each initiation-event, who has responsibility:

$$\frac{\alpha}{\alpha + \text{Others}},$$

and the other IDUs present each have responsibility

$$\frac{1}{\alpha + \text{Others}}.$$

By definition, the total responsibility then sums to one for each initiation:

$$\frac{\alpha}{\alpha + \text{Others}} + \underbrace{\frac{1}{\alpha + \text{Others}} + \cdots + \frac{1}{\alpha + \text{Others}}}_{\text{Number of Others present}} = \frac{\alpha}{\alpha + \text{Others}} + \frac{\text{Others}}{\alpha + \text{Others}} = 1$$

In these expressions, α, determines how much the primary initiator's responsibility is up-weighted compared to the other IDUs present, and may take any non-negative value. If α=1, we revert to equally-shared responsibility as used in the main paper. For our

example of non-equally-shared responsibility above we used α = 3. If α is very large, then the co-present IDUs will have near zero responsibility.

For each initiation-event, one of the IDUs present will be the primary initiator as defined above. Assuming a NESI respondent is equally likely to be the primary initiator as one of the others co-present, then the expected EFR-rate would be:

$$\begin{aligned}\lambda_{EFR} &= \text{E[E[(Responsibility)|(Type of initiator)]]} \\ &= \text{(Responsibility if Primary)}P\text{(Primary)} \\ &\qquad + \text{(Responsibility if not Primary)}P\text{(not Primary)} \\ &= \frac{\alpha}{\alpha + \text{Others}}\left(\frac{1}{1+\text{Others}}\right) + \frac{1}{\alpha + \text{Others}}\left(\frac{\text{Others}}{1+\text{Others}}\right) \\ &= \frac{1}{1+\text{Others}}\end{aligned}$$

That is, if we assume that the NESI respondent will be the primary initiator an equal proportion of the time as for the others co-present, taking the expectation (averaging) returns us to our natural assumption of equally-shared responsibility among all those present, irrespective of the choice of α.
